# Evaluation of the consequences associated with diffuse vascular disease history in patients diagnosed with peripheral arterial disease: estimates from Saskatchewan health data

**DOI:** 10.1186/1471-2261-10-40

**Published:** 2010-09-02

**Authors:** Kristen Migliaccio-Walle, Michael Stokes, Irina Proskorovsky, Dan Popovici-Toma, Wissam El-Hadi

**Affiliations:** 1 United BioSource Corporation, Lexington, MA, USA; 2 United BioSource Corporation, Montréal, QC, Canada; 3 sanofi-aventis Canada Inc., Laval, QC, Canada; 4 Bristol-Myers Squibb Canada, St-Laurent, QC, Canada

## Abstract

**Background:**

Peripheral arterial disease (PAD) is caused by narrowing of the arteries in the lower extremities. Limited data exist concerning the impact of diffuse vascular disease (DVD) on prognosis and costs. Thus, the objective of this study is to estimate the impact of DVD on morbidity, mortality and costs.

**Methods:**

PAD was identified between 1985 and 1995 and classified by extent of DVD at diagnosis: none (PAD only, reference group), prior myocardial infarction (MI), prior stroke, prior MI *and *stroke (MI + stroke), prior transient ischemic attack (TIA). Deaths and hospitalizations were identified through December 2000. Hospitalization costs were estimated from the Ontario Case Cost Project, reported in 2002 $CAD. Proportional hazards analyses measured the impact of vascular involvement on mortality while controlling for risk factors (e.g., age, cardiovascular history).

**Results:**

Overall, 16,439 patients with PAD were included; 14.8% had a prior MI, 10.2% a prior stroke, 2.6% prior MI + stroke, 6.4% prior TIA, two-thirds had PAD only. Median survival was shorter for patients with prior MI (9.3 yrs), TIA (6.3), stroke (4.7), and MI+stroke (4.1) versus the reference group (9.9, p < 0.05, all comparisons). Analyses revealed that the death risk was 60% higher in patients with prior stroke and 84% higher for MI + stroke. Atherothrombotic and bleeding event-related costs were $712, $337, $268, and $170 higher per patient/year of follow-up in patients with a history of MI+stroke, MI, stroke, and TIA, respectively.

**Conclusion:**

Patients diagnosed with PAD with DVD have higher risk of poor outcomes and increased costs.

## Background

Cardiovascular disease (CVD) risk has been studied mainly in relation to a patient's most recent event. Most secondary prevention clinical trials enroll patients at an arbitrary point in their disease. In CAPRIE, randomization was according to the most recent manifestation of CVD, yet fully understanding a new therapy's impact requires understanding the impact of involvement in multiple vascular beds on subsequent risk. Peripheral arterial disease (PAD) is a condition caused by progressive narrowing of the arteries in the lower extremities [1] often leading to serious cardiovascular complications and, sometimes, death [2-6]. Studies conducted in the United States and Europe have shown that the prevalence of PAD ranges from <5% in younger age groups to >20% in the elderly [7-9]. In Canada, the prevalence of PAD is estimated around 4% in people ≥40 years of age [1].

Some evidence indicates that PAD negatively impacts the prognosis of patients with diffuse vascular disease (DVD). Studies have demonstrated that history of PAD is associated with more extensive coronary artery disease (CAD) and higher death rates [3,10] in patients suffering a coronary artery event. Moreover, evidence shows that this subgroup is often under-diagnosed and frequently undertreated; only 25% of patients diagnosed with PAD receive treatment despite having an increased risk for poor outcomes [9,11]. Limited data examining the impact of diffuse vascular involvement (>1 vascular bed) on outcomes and survival following a diagnosis of PAD are available. This study examines the relationship using data from Saskatchewan Health.

## Methods

### Data Source & Patient Population

Data for this study were obtained from Saskatchewan Health, an agency responsible for healthcare administration in the Canadian province of Saskatchewan. Saskatchewan Health maintains 10 databases tracking all formulary outpatient prescriptions, physician services, hospitalizations, and vital statistics for the approximately 1 million residents covered by provincial health insurance [12].

Residents of Saskatchewan ≥21 years of age with a diagnosis of PAD between January 1, 1985 and December 31, 1995 were selected for inclusion. We identified patients with PAD using the International Classification of Diseases, 9^th ^Revision (ICD-9) diagnosis codes 440.x (atherosclerosis), 440.2 (atherosclerosis of native arteries of the extremities), and 443.9 (peripheral vascular disease (PVD)). Only the truncated three-digit code was available for diagnoses made on physician visit, thus patients with the broader three-digit code 443 (other PVD) were included if they received a prescription for pentoxifylline at any point in time as this was considered a conclusive clinical indicator of PAD. No exclusion criteria were specified. The date of the first PAD diagnosis on either a hospital *or *physician services record was taken as the study index date.

Patients were classified into one of five subgroups according to of the extent of DVD involvement at index diagnosis: (1) no history of DVD (*PAD only*); (2) history of myocardial infarction (MI) only (*MI*); (3) history of stroke only (*stroke*); (4) history of MI and stroke (*MI+stroke*); and (5) history of transient ischemic attack (TIA) only (*TIA*). This was ascertained using ICD-9 diagnosis codes from hospital and physician services records (ischemic stroke: 433.x, 434.x; TIA: 435.9, 436.x; MI/unstable angina: 410.x, 412.x). Medical histories were available back to January 1, 1980. Histories were also examined to identify other comorbid conditions including atrial fibrillation, angina, heart failure, hypercholesterolemia, hypertension, and diabetes. The location (hospital or physician visit) where the index PAD diagnosis occurred was also recorded.

### Hospitalizations Related to Atherothrombotic Disease

Morbidity was assessed by examining the hospitalizations for 1) any cause and 2) those related to major atherothrombotic and bleeding events (ABE). ABE-related hospitalizations were classified using primary ICD-9 diagnosis codes appearing on hospital records. Hospitalizations were considered ABE-related if they were coded with a diagnosis code indicating ischemic stroke (433.x, 434.x), TIA (436.x, 435.9), MI (410.x, 412.x), angina (411.x, 413.x), heart failure (428.x), PAD (440.x, 443.x), or bleeding (430.x, 431.x, 432.x, 459.x, 578.x). The time to first hospitalization (any cause or ABE-related) was calculated in years from the study index date until the first hospitalization event. The time in years from the first to second study hospitalization (any cause or ABE-related) was also assessed.

### Hospitalization Costs Related to Atherothrombotic Disease

Hospital cost data were not obtained from Saskatchewan Health for this study. Thus, costs related to ABE events were acquired from an external source--the 2000 Ontario Case Cost Project (OCCP) Case Mix Group Costing Analysis Tool (CAT) [13]. This same approach was utilized in a prior analysis of the Saskatchewan data [14]. Studies estimating PAD costs have shown that 75% of total costs are driven by hospitalizations [15]. A listing of the cost estimates used for specific types of ABE-related hospitalizations is shown in Table 1. Hospital costs are standardized to 2002 $CAD and presented as per-patient means.

**Table 1:** OCCP Case Mix Group cost estimates

Hospitalization type	Costs (2002 $CAD)
**Cerebrovascular**	
Ischemic stroke	$7,793
TIA with no related procedures	$2,687
TIA with carotid endarterectomy	$4,689
**Cardiovascular**	
*AMI with no surgical procedures*	
AMI, unspecified cardiac condition	$3,805
AMI with CHF	$7,064
AMI with angina	$4,564
AMI with cardiogenic shock and/or PE	$16,252
*AMI with surgical procedures*	
AMI with CABG and CC	$18,564
AMI with CABG	$12,004
AMI with PCTA	$8,189
AMI with CC/angiography	$6,161
AMI with CHF and CC/angiography	$9,349
AMI with angina and CC/angiography	$6,661
AMI with permanent pacemaker	$7,379
*Angina*	
Angina as primary diagnosis	$2,352
UA with cardiac complication	$3,227
UA without cardiac complication	$2,144
UA with cardiogenic shock and/or PE	$16,253
*Angina with surgical procedures*	
UA or angina with CABG and CC	$18,564
UA or angina with CABG	$12,004
Angina w/o cardiac complication with PTCA or stent	$4,748
Angina with cardiac complication with PTCA or stent	$8,189
UA with CC/coronary angio	$5,803
UA with cardiac complications and CC/coronary angio	$8,774
Angina with CC/coronary angio	$3,740
*Other cardiac problems*	
CHF	$4,074
CHF with CC	$4,916
Arrhythmia/Conduction disorders no pacemaker	$2,159
Arrhythmia/Conduction disorders with permanent pacemaker	$7,379
*Peripheral Vascular Disease*	
PAD, no major vascular surgery	$4,273
PAD with aorto-iliac-femoral bypass graft	$11,403
PAD with intra-abdominal bypass graft	$11,403
PAD with other peripheral bypass graft	$4,017
PAD with lower limb Embolectomy	$4,017
PAD with upper limb or toe amputation	$9,239
PAD with foot amputation	$12,519
PAD with below the knee amputation	$12,344
PAD with above the knee amputation	$12,420
*Major bleeds*	
ICH, no surgical procedures	$12,134
GI bleed, no surgical procedures	$2,826
Hemorrhage, unspecified with no surgical procedures	$6,218
GI bleed with gastrectomy/other major gastric surgery	$11,050
GI bleed with endoscopy	$4,017

AMI = acute myocardial infarction

CABG = coronary artery bypass graft

CC = cardiac catheterization

CHF = congestive heart failure

GI = gastrointestinal

ICH = intracranial hemorrhage

PAD = peripheral arterial disease

PE = pulmonary embolus

PTCA = percutaneous transluminal coronary angioplasty

TIA = transient ischemic attack

UA = unstable angina

### Study Follow-up

Study outcomes including survival, morbidity, and hospital costs were evaluated from the index PAD diagnosis and ending at the earlier of the date of death, study conclusion (December 31, 2000), or end of follow-up in the Saskatchewan data.

### Data Analyses

Baseline demographic and clinical characteristics including age, gender, index location (i.e., hospital or physician record), and medical history were compared across subgroups. Group comparisons were made using two-sided Pearson chi square and *t*-test statistics for categorical and continuous measures, respectively.

Life expectancy for each vascular involvement subgroup was estimated using Kaplan-Meier survival analysis techniques. Patient time was calculated as the number of months from the index PAD diagnosis until either the date of death or censoring. Patients were censored if they were still alive as of their last day of available follow-up in the data set. Overall survival was compared using the log-rank test. Proportional hazards models were created to measure the impact of DVD history on mortality while controlling for competing risk factors including age, gender, and past histories of atrial fibrillation, angina, heart failure, and hypertension. Interaction terms for history of TIA and time, history of MI+stroke and time, history of MI and time, as well as history of stroke and time were included because the proportionality of the effect of these determinants could not be verified.

The mean time to the first hospitalization and mean time from the first to the second hospitalization was determined for any cause and ABE-related hospitalizations. Only patients who experienced a hospitalization contributed to the mean time calculations, therefore, comparisons between the groups were made using the t-test statistic. The proportion of patients hospitalized for specific ABE-related events (Stroke/TIA, MI/Angina, and Other CVD) were compared across subgroups. Group comparisons were made using the two-sided Pearson chi square test.

Cost data were analyzed using descriptive statistics (i.e., means and standard deviations). Non-parametric bootstrap methods were used to calculate the 95% confidence intervals around the per-patient cost estimates [16]. First, the data were re-sampled with replacement from the study dataset to create 1,000 random samples. Next, inpatient ABE-related costs were calculated for each of the 1,000 study replicates. Finally, confidence intervals were derived from the sample distribution of costs of the 1,000 replicates at the 2.5% and 97.5% quantiles. Costs were also calculated per study year to adjust for differences in the length of follow-up observed between study groups. Statistical significance was evaluated at an alpha = 0.05 for all tests. Unless otherwise specified, a *t-*test was performed using the PAD only subgroup as the reference category.

## Results

### Patient Characteristics

Additional file 1: Table S1 displays baseline characteristics by vascular disease subgroup. A cohort of 16,439 patients diagnosed with PAD was identified including 2,437 (14.8%) with a prior MI, 1,679 (10.2%) with prior stroke, 431 (2.6%) with prior MI + stroke, and 1,048 (6.4%) with prior TIA. Patients across any of the subgroups containing stroke/TIA were almost 4 years older than those with PAD only (70.3 vs. 66.7 years, p < 0.001) and MI only (70.3 vs. 65.6 years, p < 0.001). Patients with DVD were more likely to have ABE-related comorbidities (90% vs. 73%, p < 0.001). Length of follow-up was on average one year shorter in patients with a history of DVD (6.4 vs. 7.4 years, p < 0.001).

### Mortality & Morbidity

**Figure 1:**
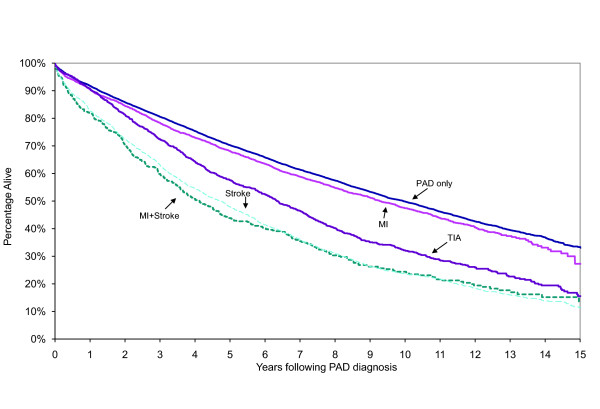
Kaplan-Meier survival following diagnosis of PAD by type of diffuse vascular disease history

Patients with a history of DVD were more likely to die during the study period (Additional file 1: Table S1; 67.6% vs. 52.3%, p < 0.001). Median survival (in years) was progressively worse for patients with a history of MI (9.3 years), TIA (6.3), stroke (4.7), and MI+stroke (4.1) compared to the PAD only subgroup (9.9) (Figure 1, p < 0.05, all comparisons). Table 2 displays results of proportional hazards models examining the risk of death, subsequent MI, and subsequent ischemic stroke. After controlling for competing risk factors, a 60% and 84% increase in the hazard of death in patients with a history of stroke (p < 0.001) and MI+stroke (p < 0.001), respectively, was observed. Diffuse involvement including a stroke or MI+stroke was also associated with higher rates of MI (Stroke: p < 0.05, MI+stroke: p < 0.001) and ischemic stroke (Stroke: p < 0.001, MI+stroke: p < 0.001). Those with a history of TIA were 61% more likely to have an ischemic stroke (p < 0. 001).

**Table 2:** Hazard ratios of death, MI and ischemic stroke by risk factors

Study Measure	Death	MI	Ischemic stroke
Age at index diagnosis	1.08^†^	1.02^†^	1.05^†^
Male gender	1.28^†^	1.69^†^	1.32^†^
History of:			
MI	1.06**	1.66^†^	1.17*
Stroke	1.60^†^	1.29*	3.11^†^
MI+stroke	1.84^†^	1.76^†^	3.26^†^
Atrial fibrillation	1.17**	--	1.64^†^
Angina	0.84^†^	1.44^†^	--
Heart failure	1.89^†^	--	1.18*
Hypercholesterolemia	0.77^†^	--	--
Hypertension	--	1.21**	1.52^†^
Diabetes	1.37^†^	1.49^†^	--
TIA	1.04	--	1.61^†^
Interaction terms			
TIA × follow-up	1.02*	--	--
MIx follow-up	--	--	--
Stroke × follow-up	--	--	0.95*
MI + stroke × follow-up	0.96*	--	0.93*

Final models are presented; factors that were not statistically significant were excluded since they can introduce noise and reduce precision of estimates

MI = myocardial infarction

TIA = transient ischemic attack

*p < 0.05

**p < 0.01

^† ^p < 0.001

-- = not statistically significant

### Hospitalizations Related to Atherothrombotic Disease

**Figure 2:**
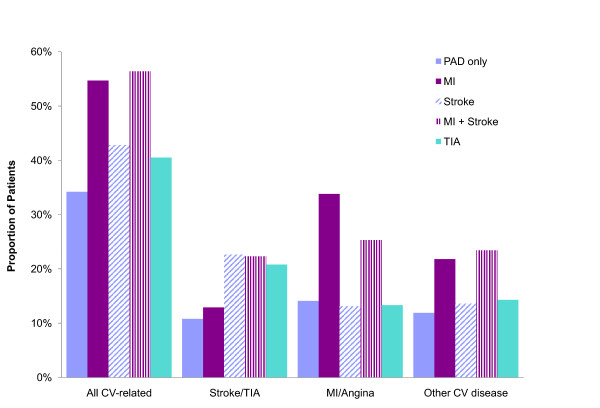
Proportion of patients hospitalized for CV-related events by diffuse vascular disease history

A higher proportion of patients with DVD were hospitalized for ABE-related events versus PAD only (Figure 2; 48.6% vs. 34.2%, p < 0.001). The rate of hospitalization for ABE events increased according to the type and extent of DVD (Figure 2, TIA: 40.5%, Stroke: 42.8%, MI: 54.5%, and MI + Stroke: 56.4%). Patients with a history of MI were more likely to be hospitalized for an MI/angina event compared to those with PAD only (33.8% vs. 14.1%, p < 0.001) or those with a history of stroke or TIA (33.8% vs. 14.8%, p < 0.001). Patients with a medical history of stroke or TIA were also more susceptible to having a stroke or TIA event requiring hospitalization compared to patients without such a history (22.0% vs. 10.8%, p < 0.001).

The average time until a first ABE-related hospitalization was almost one year shorter for patients with DVD compared to PAD only (Additional file 2: Table S2, 4.4 vs. 5.3 years, p < 0.001). Although patients with a history of DVD were hospitalized for any cause at similar rates compared to PAD only (86%), the average time to this hospitalization was 6 months shorter for patients with DVD as compared to those with PAD only (Additional file 2: Table S2, 1.7 vs. 2.3 years, p < 0.001). Patients with a history of DVD were also more likely to suffer a repeat ABE-related hospitalization compared with PAD only patients (25.8% vs. 15.6%, p < 0.001). Amongst patients who suffered a second event, however, the length of time from the first to second ABE-related hospitalization in patients with a history of DVD compared with those diagnosed with PAD only was not as disparate as the time to the first ABE-related hospitalization (DVD: 1.7 vs. PAD only: 1.9 years, p = 0.02). The proportion of patients with a second hospitalization from any cause was comparable in those with DVD (72.2%) and PAD only (69.3%, p < 0.01). Patients with a history of DVD had a second hospitalization from any cause that occurred on average 3.6 months earlier versus PAD only (1.3 vs. 1.6 years, p < 0.001).

### Hospitalization Costs Related to Atherothrombotic Disease

**Figure 3:**
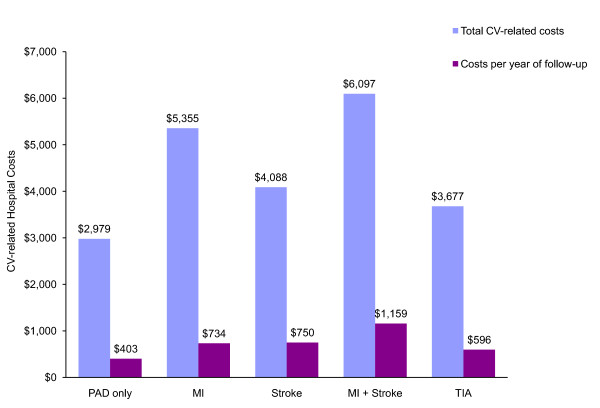
Mean costs of hospitalizations related to cardiovascular events (CV), by type of diffuse vascular history

The patient subgroups with a history of DVD had higher mean per-patient ABE-related hospital costs (DVD: $4 718, 95% CI: $3 391-$6 611, p < 0.0001 vs. PAD only; MI: $5 355, 95% CI: $5 088-$6 753, p < 0.0001; Stroke: $4 088, 95% CI: $3 774-$4 400, p < 0.0001; MI+Stroke: $6 097, 95% CI: $5 372-$6 884, p < 0.0001; TIA: $3 677, 95% CI: $3 248-$4 159, p < 0.002) in comparison to PAD only ($2 979, 95% CI: $2 854-$3 102) (Figure 3). Inpatient ABE-related per-patient costs were $757, $348, $331, and $193 higher per year of follow-up versus PAD only in patients with a history of MI+stroke, stroke, MI, and TIA, respectively (Figure 3).

## Discussion

This study presents, to the best of our knowledge, a novel evaluation of the impact of DVD involvement on outcomes in patients diagnosed with PAD. Data from the Saskatchewan Health databases were used to examine the impact of varying degrees of involvement on survival, hospitalizations, and costs following a diagnosis of PAD.

Previous studies have focused on the link between a prior PAD diagnosis and higher mortality in patients with CAD [3,10,17]. These results can be compared to those reported in these prior studies to understand the difference in impact that a prior diagnosis of PAD has on mortality in CAD versus the impact that a prior DVD diagnosis has on patients with PAD. Cotter *et al. *found that, after adjustment for risk factors, a history of cerebrovascular accidents, TIA, or PAD in patients with acute coronary syndromes is significantly associated with a 47% increase in the risk of mortality [10]. A study investigating the long-term impact of PAD on outcomes in patients with acute MI (AMI) found that the risk of death was 29% higher in patients with prior PAD [17]. Froehlich and colleagues found that a prior diagnosis of PAD increases the risk of mortality by nearly 19% in patients with acute coronary syndromes [3]. In the current study, history of stroke or MI+stroke had a greater impact on mortality in patients with PAD compared to the reported impact of prior PAD in patients with CAD [3]. It could be posited that the occurrence of an acute event prior to, rather than following, diagnosis of PAD is an indicator of more severe disease. Prior MI in patients with PAD had little added impact on mortality whereas the impact of prior PAD in patients with AMI has been shown to be significant [17].

That patients with PAD continue to be under-diagnosed and under-managed despite the pervasive evidence that they are at increased risk for poor outcomes [3,9-11] is astounding. Our analyses further contribute to this knowledge base indicating that patients with PAD are at considerable risk for morbidity, mortality, and incurring substantial costs associated with these. Moreover, when there is a history of DVD these patients face an even higher burden in terms of subsequent events and costs. Limited data examining the impact of diffuse vascular involvement (>1 vascular bed) on outcomes and survival following a diagnosis of PAD are available.

This study was subject to several limitations. In identifying the PAD population, study subgroups, and ABE-related hospitalizations, we relied on ICD-9 diagnosis codes submitted by health-care providers to the province of Saskatchewan. These codes are primarily used for administrative purposes in obtaining reimbursement for the services provided to Saskatchewan residents, thus research is not the primary purpose of these data. Therefore, there is the possibility that some PAD patients and ABE-related hospitalizations were misclassified. Past validation studies examining the accuracy of the administrative codes contained within the Saskatchewan data, however, have revealed low error rates [12].

In our analysis of costs, only measures related to ABE hospitalizations were considered thus not accounting for the additional costs associated with outpatient management and medication use. However, prior studies examining PAD have shown that 75% of total costs are driven by hospitalizations [15]. Therefore, we believe that while this approach may underestimate the absolute values, it nevertheless provides a reasonable estimate of the relative economic burden of PAD. Hospital costs were estimated from the province of Ontario using the 1999/2000 OCCP Case Mix Group CAT, a source external to the Saskatchewan data. Hospitalization data could not be obtained with consistent reliability from Saskatchewan at the time of this study and therefore it is not clear the extent to which the Ontario rates reflect the reimbursement rates provided to hospitals from Saskatchewan.

## Conclusion

This study indicates that patients with DVD involvement have significantly poorer prognoses and higher costs and resource use as compared to patients diagnosed only with PAD. A history of DVD amongst these patients, particularly MI+stroke, increased the risk of morbidity and mortality and the corresponding costs. Appropriate management of patients with PAD is an important public health issue as this condition affects a large number of Canadians with involvement of DVD being present in one-third of our actual practice population. Amongst the Canadian population in the REACH Registry, over 60% of the PAD patients have DVD [18]. When compared to the Saskatchewan database, a broader range of patients were enrolled in the REACH Registry. Thus, PAD identified and treated early, before vascular involvement becomes diffuse, provides the opportunity to significantly reduce morbidity and mortality amongst this patient group. Moreover, it is critical to understand the full extent of vascular disease involvement when determining the prognosis and management of patients who present with PAD.

## List Of Abbreviations

ABE: atherothrombotic and bleeding event; AMI: acute myocardial infarction; CAD: coronary artery disease; CAT: Costing Analysis Tool; CVD: cardiovascular disease; DVD: diffuse vascular disease; ICD-9: International Classification of Diseases, 9th Revision; MI: myocardial infarction; OCCP: Ontario Case Cost Project; PAD: peripheral arterial disease; PVD: peripheral vascular disease; TIA: transient ischemic attack

## Competing interests

Funding for this research was provided by Bristol-Myers Squibb Canada and sanofi-aventis Canada. Ms. Migliaccio-Walle, Mr. Stokes and Ms. Proskorovsky are affiliated with United BioSource Corporation. Dr. Popovici-Toma and Mr. El-Hadi are affiliated with sanofi-aventis Canada Inc. and Bristol-Myers Squibb Canada, respectively.

## Authors' contributions

KM was the lead scientist on the design and conceptualization of this study and contributed to the writing and interpretation of results. MS participated in the writing of the manuscript and preparation and interpretation of results. IP participated in the design of the study and carried out all of the statistical analyses. She also assisted in the interpretation of results. DP and WE contributed to the conceptualization of this study and to the interpretation of results. All authors read and approved the final manuscript.

## Pre-publication history

The pre-publication history for this paper can be accessed here:

http://www.biomedcentral.com/1471-2261/10/40/prepub

## Supplementary Material

Additional file 1 **Table S1. Patient characteristics at index diagnosis by diffuse vascular disease history**. Table. Click here for file

Additional file 2 **Table S2. Intervals between hospitalizations by type of diffuse vascular disease history**. Table. Click here for file
