# Supplementary material for: Evaluation of the consequences associated with diffuse vascular disease history in patients diagnosed with peripheral arterial disease: estimates from Saskatchewan health data
Source: BMC Cardiovasc Disord. 2010 Sep 2;10:40. doi: 10.1186/1471-2261-10-40 (PMC2940788; doi:10.1186/1471-2261-10-40)
Supplement: Additional file 1 — Table S1. Patient characteristics at index diagnosis by diffuse vascular disease history. Table. [file 1471-2261-10-40-S1.DOCX]

| **Additional file 1: Table S1. Patient characteristics at index diagnosis by diffuse vascular disease history** | | | | | | | | | | |
| --- | --- | --- | --- | --- | --- | --- | --- | --- | --- | --- |
| **Study Measure** | **PAD only**  **(reference group)** | | **MI** | | **Stroke** | | **MI+Stroke** | | **TIA** | |
| Number of patients (n) | 10,844 | 66.0% | 2,437 | 14.8% | 1,679 | 10.2% | 431 | 2.6% | 1,048 | 6.4% |
| Age, years |  |  |  |  |  |  |  |  |  |  |
| Mean | 66.7 | | 65.6^†^ | | 69.9^†^ | | 69.4^†^ | | 71.4^†^ | |
| SD | 9.8 | | 9.3 | | 5.7 | | 6.3 | | 5.2 | |
| Male, n (%) | 5,548 | (51.2) | 1734^†^ | (71.2) | 920** | (54.8) | 300^†^ | (69.6) | 527 | (50.3) |
| Index PAD diagnosis from hospital record, n (%) | 1,047 | (9.7) | 225 | (9.2) | 225^†^ | (13.4) | 65** | (15.1) | 97 | (9.3) |
| Medical history, n (%) |  | |  | |  | |  | |  |  |
| Atrial fibrillation | 442 | (4.1) | 179^†^ | (7.4) | 116^†^ | (6.9) | 51^†^ | (11.8) | 74^†^ | (7.1) |
| Angina | 2,589 | (23.9) | 1843^†^ | (75.6) | 437 | (26.0) | 280^†^ | (65.0) | 297** | (28.3) |
| Heart failure | 2,158 | (19.9) | 881^†^ | (36.2) | 589^†^ | (35.1) | 200^†^ | (46.4) | 349^†^ | (33.3) |
| Hypercholesterolemia | 673 | (6.2) | 264^†^ | (10.8) | 89 | (5.3) | 48^†^ | (11.1) | 54 | (5.2) |
| Hypertension | 5,879 | (54.2) | 1506^†^ | (61.8) | 1130^†^ | (67.3) | 320^†^ | (74.3) | 719^†^ | (68.6) |
| Diabetes | 1,922 | (17.7) | 559^†^ | (22.9) | 410^†^ | (24.4) | 98** | (22.7) | 183 | (17.5) |
| At least one condition | 7,879 | (72.7) | 2290^†^ | (94.0) | 1441^†^ | (85.8) | 404^†^ | (93.7) | 875^†^ | (83.5) |
| Died during study period, n (%) | 5,713 | (52.7) | 1371** | (56.3) | 1342^†^ | (79.9) | 338^†^ | (78.4) | 730^†^ | (69.7) |
| Follow-up, years |  |  |  |  |  |  |  |  |  |  |
| Mean | 7.4 | | 7.3 | | 5.45^†^ | | 5.26^†^ | | 6.17^†^ | |
| SD | 4.5 | | 4.4 | | 4.4 | | 4.3 | | 4.2 | |
| MI = myocardial infarction *p<0.05  PAD = peripheral arterial disease **p<0.01  TIA = transient ischemic attack ^†^p<0.001  SD = standard deviation | | | | | | | | | | |
