# Supplementary material for: Evaluation of the consequences associated with diffuse vascular disease history in patients diagnosed with peripheral arterial disease: estimates from Saskatchewan health data
Source: BMC Cardiovasc Disord. 2010 Sep 2;10:40. doi: 10.1186/1471-2261-10-40 (PMC2940788; doi:10.1186/1471-2261-10-40)
Supplement: Additional file 2 — Table S2. Intervals between hospitalizations by type of diffuse vascular disease history. Table. [file 1471-2261-10-40-S2.DOCX]

| **Additional file 2: Table S2. Intervals between hospitalizations by type of diffuse vascular disease history** | | | | | | | |
| --- | --- | --- | --- | --- | --- | --- | --- |
| **Study Measure** | **Number** | **Mean Years (SD)** | **Minimum** | **25th percentile** | **Median** | **75th percentile** | **Maximum** |
| **Any cause hospitalization** |  |  |  |  |  |  |  |
| Time to first hospitalization |  |  |  |  |  |  |  |
| PAD Only (reference group) | 9,273 | 2.3 (2.8) | 0 | 0.26 | 1.11 | 3.22 | 15.22 |
| MI | 2,194 | 1.7 (2.4)^†^ | 0 | 0.13 | 0.62 | 2.25 | 14.08 |
| Stroke | 1,356 | 1.6 (2.2)^†^ | 0 | 0.23 | 0.72 | 2.01 | 13.48 |
| MI+Stroke | 380 | 1.4 (2.1)^†^ | 0.01 | 0.2 | 0.64 | 1.58 | 13.25 |
| TIA | 902 | 1.8 (2.2)^†^ | 0 | 0.27 | 0.92 | 2.31 | 15.64 |
| Time to second hospitalization |  |  |  |  |  |  |  |
| PAD Only | 7,518 | 1.6 (2.1) | 0 | 0.2 | 0.72 | 2.11 | 13.18 |
| MI | 1,913 | 1.5 (2.1) | 0.01 | 0.15 | 0.57 | 1.9 | 13.08 |
| Stroke | 306 | 1.3 (1.7)* | 0.01 | 0.18 | 0.66 | 1.7 | 14.89 |
| MI+Stroke | 1,075 | 1.0 (1.6)^†^ | 0.01 | 0.14 | 0.42 | 1.24 | 10.35 |
| TIA | 743 | 1.2 (1.5)^†^ | 0.01 | 0.18 | 0.57 | 1.61 | 11.53 |
| **Hospitalization for CV event** |  |  |  |  |  |  |  |
| Time to first hospitalization |  |  |  |  |  |  |  |
| PAD Only | 3,704 | 5.3 (4.0) | 0 | 1.71 | 4.67 | 8.12 | 15.93 |
| MI | 1,332 | 4.6 (3.8)^†^ | 0 | 1.19 | 3.71 | 7.53 | 15.31 |
| Stroke | 719 | 4.2 (3.6)^†^ | 0.01 | 1.11 | 3.12 | 6.37 | 15.68 |
| MI+Stroke | 243 | 3.4 (3.2)^†^ | 0.01 | 0.76 | 2.42 | 4.85 | 13.97 |
| TIA | 424 | 4.5 (3.6)^†^ | 0.01 | 1.56 | 3.69 | 6.66 | 15.59 |
| Time to second hospitalization |  |  |  |  |  |  |  |
| PAD Only | 1,696 | 1.9 (2.3) | 0.01 | 0.25 | 0.93 | 2.61 | 13.81 |
| MI | 777 | 1.7 (2.2)* | 0 | 0.22 | 0.8 | 2.44 | 13.19 |
| Stroke | 137 | 1.7 (2.3) | 0 | 0.23 | 0.77 | 2.13 | 13.37 |
| MI+Stroke | 325 | 1.6 (2.2)* | 0.01 | 0.23 | 0.75 | 2.13 | 12.69 |
| TIA | 205 | 1.5 (1.9)* | 0.01 | 0.28 | 0.77 | 2.11 | 11.53 |
